# Observations on the Peripneumonia Typhodes, Now Prevailing in Several Districts of the United States. In a Letter to T. Romeyn Beck, M.D. &c. Albany, from David Hosack, M.D. &c.

**Published:** 1814-06

**Authors:** David Hosack


*For the Medical and Physical Journal.*

### *Observations on the Peripneumonia Typhodes, now prevailing in several Districts of the United States. In a Letter to* T. Romeyn Beck, M.D. &c. *Albany, from* David Hosack, M.D. &c.

DEAR SIR, *New-York, Feb.* 3, 1813.

AS far as you have been enabled to procure information from *books*, you are probably well acquainted with the nature of the fatal epidemic which at present prevails in the city of Albany; but as you have been only a short time in practice, this is probably the first opportunity you have had of of becoming practically conversant with this disease. I therefore take the liberty of communicating to you such remarks as the cases I have occasionally seen in this city and in the county of Westchester have suggested.

The disease at present prevailing, you know, is not a new disease, as it has been hastily represented by some physicians: on the contrary, it has been well described by Sauvages, Huxham, and others; by the former under the very appropriate appellation of "*Peripneumonia typhodes.*"* Nor is this a new disease in the United States: in the first volume of the Medical and Philosophical Register you will see a very valuable paper upon this subject by the late Dr. John Bard, in which he describes a similar epidemic which prevailed on Long-Island in the winter of 1749, and which was then known by the name of the "*Malignant Pleurisy.*"

In the second volume of the Medical Repository (first series) the same disease is noticed by Dr. Hugh Williamson, as it prevailed in North Carolina, in the year 1792. In the southern states it is commonly called, "*Pleurisy in the Head,*" in consequence of the violent pain in the head, which frequently attends the disease in that climate.

Malignant pleurisy, or rather *Typhus Peripneumony*, well expresses the mixed character of this disease; for at the same time that it is attended with inflammation of the lungs, and, in some instances, with inflammation of the brain, the general affection of the whole system is certainly that of *typhus fever.*

That inflammation of the lungs frequently constitutes a part of the disease, is manifest, not only from the presence of those symptoms usually attendant upon pneumonic inflammation, viz. cough, pain in the chest, especially upon taking a full inspiration, expectoration tinged with blood in the early stage of the complaint; but it is also evident from the phenomena presented upon an examination of the body after death: the overloaded state of the vessels of the lungs, the large effusions of serum and sometimes purulent matter, the adhesions found between the membranes covering the lungs, and those lining the chest, all clearly show that the patient has been destroyed by such inflammation.† In like manner, in some cases the whole force of the disease is vented upon the brain, producing similar phenomena in that

* See his Nosologia Methodica, vol. i. For other synonyms, see also Cullen's Nosology, under the head of "Peripneumoniæ idiopathicæ complicatæ febre," vol. ii. p. 101.

† See Report of the Mass. Med. Society, and Dr. Hudson's letter on the prevailing epidemic.

 organ.

organ. On the other hand the usual symptoms of a putrescent state of body, the petechiæ, blotches, hemorrhages in the latter stage of the disease, the offensive state of the excretions in general, and great prostration of the powers of life, which rapidly ensues, no less declare the enfeebled and vitiated state of the whole habit. You have therefore two opposite conditions of body to contend with: *local inflammation* on the one hand, a *typhus state of the whole system* on the other.

The causes of the disease are no less compounded than the disease itself. The local inflammatory affections are probably occasioned by the *sensible* changes of the atmosphere, while the typhoid character of the disease is derived from an *epidemic constitution of the air*, the same which has given rise to the *typhus petechialis*, or *spotted fever*, which has prevailed for some time past in our northern and eastern states, and which is doubtless the same disease as that now prevailing in Albany, with the exception, that the present epidemic is complicated with the symptoms of local inflammation of the chest, brain, throat, &c. the effect of the present cold season of the year. With this view of the *mixed* nature of the disease, and of the combined causes which have produced it, we are prepared to expect the various and opposite opinions and modes of practice which have been adopted by different physicians. We accordingly find some prescribing the strict antiphlogistic treatment by large and repeated blood-letting, active cathartics, and other depleting remedies, treating the disease as purely inflammatory.

On the other hand, we find another class of practitioners pursuing the opposite course of exciting the system by the most powerful and diffusible stimuli, to counteract the putrescent state of body, alleging, that it is exclusively a putrid disease, and only to be controuled by antiseptics and the avoidance of all those means which are calculated to debilitate the system. As far as I have seen the disease, they are both wrong: the indiscriminate use of the lancet recommended by some, is, in my opinion, an additional source of the mortality of the disease. On the other hand, the practice of administering brandy and other ardent spirits in the quantity they have been lately prescribed, is truly adding fire to the flame that is already consuming the patient, and cannot be justified either by principle or practice. But the prudent physician will avoid both Scylla and Charybdis. In the young and athletic, he will prevent the brain from being inundated with blood, by the early and judicious use of the lancet, blisters, and other means usually prescribed for diminishing inflammation, keeping in view the age, strength, constitution of his patient, and the general symptoms indicating a putrescent state of the system. On the contrary, in feeble old age, in the habit debilitated by disease, or intemperance, in which those inflammatory symptoms are less violent, and the tendency to putrefaction is most predominant, he will depend chiefly on those means usually resorted to for the purpose of promoting the perspiration and other excretions; at the same time, that by suitable antiseptic drinks and nourishment, he will guard against that debility which so rapidly ensues in this condition of the system. He will in such cases, of course, carefully abstain from the use of blood-letting and other depleting remedies. But he will certainly not effect the first purpose by the excessive use of brandy and ardent spirits. So far from promoting the excretions of the system, they actually restrain those very evacuations which it should be our object to promote, and by which alone we are enabled to counteract the typhoid state of body in this or any other febrile disease.

As a substitute, therefore, for this *brandy* system in those cases where this typhoid tendency prevails, and you are forbidden the use of the lancet and other depleting remedies, or where the symptoms of local inflammation are so mild that they are not indicated, let me recommend to you, after emptying the bowels by an enema or *mild* purgative, to make free use of the *warm bath*, *fomentations* of vinegar and water to the extremities, the liberal use of the infusion of *snake root*, the *eupatorium*, and wine whey in very debilitated habits, or where the powers of life are much reduced, in order thereby to procure a plentiful perspiration. By this evacuation we not only counteract the general vitiated state of the fluids, but we at the same time diminish, and in some cases totally remove the local irritation which affects the lungs or other organs, involved in the disease.

For the benefits which have been derived from the sudorific treatment, and the means of effecting it, in the petechial or spotted fever, which treatment promises to be no less beneficial in the present form of the epidemic now prevailing in your city, let me earnestly recommend to you the perusal of the very luminous report of the Massachusetts Medical Society, published in the second volume of their Medical Papers for the year 1810, and of which you will find a copious analysis in the first volume of the American Medical and Philosophical Register. I am, dear Sir, your's, &c.

*Dr. Beck.* DAVID HOSACK.

